# 1,2-dithiol-3-thione and dithioester analogues: potential radioprotectors.

**DOI:** 10.1038/bjc.1990.221

**Published:** 1990-07

**Authors:** B. A. Teicher, J. Stemwedel, T. S. Herman, P. K. Ghoshal, A. Rosowsky

**Affiliations:** Dana-Farber Cancer Institute, Boston, MA 02115.

## Abstract

Several 1,2-dithiol-3-thione and dithioester compounds were assayed for radioprotective capabilities in EMT6 cells in vitro. The 1,2-dithiol-3-thiones were generally more cytotoxic than the dithioesters and in some instances were more cytotoxic toward hypoxic cells than toward normally oxygenated cells. When the drugs were present at a concentration of 500 microM for 1 h prior to and during radiation delivery, the 5-(2-thienyl)-1,2-dithiol-3-thione produced a radiation protection factor (RPF) of 2.7 at 1 log of cell kill. The 4-methyl analogue of this same compound was, however, much less effective, producing a RPF of only 1.2. The 4-ethoxycarbonyl analogue was moderately active, producing a RPF of 1.7. The 4-methyl-5-(2-pyrazinyl)-1,2-dithiol-3-thione (Oltipraz) was least effective, yielding a RPF of only 1.1. Of the dithioesters tested, methyl 3-pyrrolidino-2-phenylpropene dithiocarboxylate produced a RPF of 2.6, methyl 3-piperidino-2-phenylpropenedithiocarboxylate a RPF of 2.7, and the corresponding 3-morpholino and 3-thiomorpholino derivatives RPF values of 2.7 and 2.9, respectively. The iodide salt of 4-ethoxycarbonyl-5-(2-thienyl)-1,2-dithiol-3-thione produced a RPF of 2.6 Methyl 3-cyclohexylamino-2-phenylpropenedithiocarboxylate was equally effective (RPF = 2.6). Finally, methyl 3-morpholino-3-thienyl-2-methylpropenedithiocarboxylate and methyl 3-morpholino-3-(2-pyrazinyl)-2-methylpropenedithiocarboxylate were less effective, producing RPF values of 2.0 and 1.6, respectively. These results demonstrate that several of these compounds are highly effective radioprotectors. In vitro and in vivo studies testing their efficacy are underway.


© Macmillan Press Ltd., 1990

### 1,2-Dithiol-3-thione and dithioester analogues: potential radioprotectors

B.A. Teicher[1], J. Stemwedel[1], T.S. Herman[1,2], P.K. Ghoshal[3] & A. Rosowsky[1]

[1]*Dana-Farber Cancer Institute, and* [2]*Joint Center for Radiation Therapy, 44 Binney Street, Boston, MA 02115, USA; and* [3]*Macrochem Corporation, Billerica, MA 01821, USA.*

**Summary** Several 1,2-dithiol-3-thione and dithioester compounds were assayed for radioprotective capabilities in EMT6 cells *in vitro*. The 1,2-dithiol-3-thiones were generally more cytotoxic than the dithioesters and in some instances were more cytotoxic toward hypoxic cells than toward normally oxygenated cells. When the drugs were present at a concentration of 500 μM for 1 h prior to and during radiation delivery, the 5-(2-thienyl)-1,2-dithiol-3-thione produced a radiation protection factor (RPF) of 2.7 at 1 log of cell kill. The 4-methyl analogue of this same compound was, however, much less effective, producing a RPF of only 1.2. The 4-ethoxycarbonyl analogue was moderately active, producing a RPF of 1.7. The 4-methyl-5-(2-pyrazinyl)-1,2-dithiol-3-thione (Oltipraz) was least effective, yielding a RPF of only 1.1. Of the dithioesters tested, methyl 3-pyrrolidino-2-phenylpropene dithiocarboxylate produced a RPF of 2.6, methyl 3-piperidino-2-phenylpropenedithiocarboxylate a RPF of 2.7, and the corresponding 3-morpholino and 3-thiomorpholino derivatives RPF values of 2.7 and 2.9, respectively. The iodide salt of 4-ethoxycarbonyl-5-(2-thienyl)-1,2-dithiol-3-thione produced a RPF of 2.6 Methyl 3-cyclohexylamino-2-phenylpropenedithiocarboxylate was equally effective (RPF = 2.6). Finally, methyl 3-morpholino-3-thienyl-2-methylpropenedithiocarboxylate and methyl 3-morpholino-3-(2-pyrazinyl)-2-methylpropenedithiocarboxylate were less effective, producing RPF values of 2.0 and 1.6, respectively. These results demonstrate that several of these compounds are highly effective radioprotectors. *In vitro* and *in vivo* studies testing their efficacy are underway.

In the search for methods to improve cancer therapy, one experimental strategy that has been somewhat successful in recent years is the use of normal tissue protectors. In the case of radiation, these protective agents may improve the therapeutic index by reducing toxicity and allowing higher doses of radiation to be delivered to a tumour in the vicinity of sensitive normal tissue. A large body of data exists on the use of thiol compounds as both radiation and chemoprotective drugs. One such agent, WR-2721 (*S*-2-(3-aminopropylamino)ethylphosphorothioic acid) (Yuhas & Storer, 1969; Yuhas *et al.*, 1977, 1982), is currently undergoing clinical trial (Constine *et al.*, 1986; Glover *et al.*, 1989). The monoethyl ester of glutathione, an uncharged analogue of the most abundant glutathione non-protein sulphydryl compound in cells, also shows potential application as a normal tissue protector (Teicher *et al.*, 1988).

In 1982, Bueding noted that treatment with the anti-schistosomal agent 4-methyl-5-(2-pyrazinyl)-1,2-dithiol-3-thione (Oltipraz) raised the cellular thiol levels in several tissues in mice (Bueding *et al.*, 1982). Oltipraz and other 1,2-dithiol-3-thiones have been shown to be potent inducers of enzymes involved in maintaining levels of reduced glutathione (De Long *et al.*, 1986; Davies *et al.*, 1987; Kensler *et al.*, 1987). Specifically, administration of Oltipraz or related compounds has been shown to produce increases in hepatic glutathione levels and glutathione-*S*-transferase activities and to provide protection from the hepatotoxicity of tetrachloromethane and acetaminophen (Ansher *et al.*, 1986; Davies *et al.*, 1987). This class of compounds also has potent anti-carcinogenic capacity providing protection against hepatic tumorigenesis from aflatoxin $B_1$ (Kensler *et al.*, 1987).

The present studies were undertaken to examine the cytotoxicity and potential of a series of 1,2-dithiol-3-thiones and related dithioesters to act as radioprotective agents in normally oxygenated and hypoxic EMT6 mouse mammary tumour cells *in vitro*. Some of these compounds proved highly effective protectors of normally oxygenated cells whereas others were fair protectors of normally oxygenated cells, but paradoxically also radiosensitised hypoxic cells.

Correspondence: B.A. Teicher.
Received 17 October 1989; and in revised form 9 January 1990.

### Materials and methods

### *Drugs*

The 1,2-dithiol-3-thiones and dithioesters shown in Table I were synthesised by the general method reported earlier (Foye *et al.*, 1987). Details of the chemistry of these compounds will be published later.

### *Cell line*

The EMT6 mammary tumour cell line has been widely used for the study of antineoplastic agents (Rockwell & Kallman, 1973; Rockwell *et al.*, 1972; Teicher *et al.*, 1981, 1985). The experiments described here were performed using asynchronous EMT6 cells in monolayers in exponential growth in Waymouth's medium supplemented with antibiotics (Grand Island Biological Co., Grand Island, NY, USA) and 15% newborn calf serum (Hyclone Laboratories, Logan, UT, USA). This cell line has a plating efficiency of 65-80% and a doubling time of 16-19 h *in vitro* (Rockwell *et al.*, 1972).

### *Drug cytotoxicity*

EMT6 cells were placed into T-25 flasks (Falcon Plastics). Each flask contained approximately $1 \times 10^6$ cells at the time of treatment. The drugs, in a small volume of sterile 0.9% phosphate-buffered saline, were added to the cells in complete medium in concentrations ranging from 5 to 500 μM.

### *Production of hypoxia*

To produce hypoxia, flasks containing cells in complete medium plus serum were fitted with rubber sleeve serum stoppers and exposed to a continuously flowing 95% nitrogen/5% $CO_2$ humidified atmosphere for 4 h at 37°C. Parallel flasks were maintained in 95% air/5% $CO_2$. At this time, the drug (0.10 ml) or vehicle (0.9% PBS, 0.10 ml) was added to the flasks by injection through the rubber stopper without disturbing the hypoxia (Teicher *et al.*, 1981; Teicher & Holden, 1987).

### *Radiation treatment*

For studies involving radiation, the flasks were placed in an insulated chamber filled with 95% nitrogen/5% $CO_2$ (hypoxic) or 95% air/5% $CO_2$ (normally oxygenated) atmo-

**Table I** Cytotoxicity of dithiolthiones and dithioesters in normally oxygenated and hypoxic EMT6 cells and radioprotective effect in normally oxygenated EMT6 cells

*Dithiolthiones*

| Compound number | $R_1$ | $R_2$ | $IC_{50}$ ($\mu M^a$) Normally oxygenated | Hypoxic | Radiation protection factor[b] |
|---|---|---|---|---|---|
| *1* |  | H– | 410 | 500 | 2.71 |
| *2* |  | $CH_3$– | 480 | 350 | 1.17 |
| *3* |  | $C_2H_5OC(=O)$— | 800 | 50 | 1.71 |
| *4* (Oltipraz) |  | $CH_3$– | 300 | 60 | 1.14 |
| *5* |  | | 350 | 1000 | 2.65 |

*Dithioesters*

| Compound number | $R_1$ | $R_2$ | $R_3$ | $IC_{50}$ ($\mu M^a$) Normally oxygenated | Hypoxic | Radiation protection factor[b] |
|---|---|---|---|---|---|---|
| *6* |  | H– |  | >>1000 | >>1000 | 2.60 |
| *7* |  | H– |  | 950 | 650 | 2.71 |
| *8* |  | H– |  | >>1000 | 800 | 2.60 |
| *9* |  | H– |  | 500 | >>1000 | 2.86 |
| *10* |  | H– |  | 350 | >>1000 | 2.57 |
| *11* |  |  | $H_3C$– | >>1000 | 200 | 1.97 |
| *12* |  |  | $H_3C$– | 480 | >>1000 | 1.57 |

[a]Cells in exponential growth were exposed to the drugs for 1 h and survival was measured by colony formation. $IC_{50}$ is the drug concentration which reduced the survival of the cells by 50%; [b]Protection factor is defined as the ratio of the X-ray dose with protector to the X-ray dose without protector at the level of 1 log of cell killing.

spheres. The cells were irradiated with a $^{137}Cs$ radiation unit (Gammacell 40, Atomic Energy of Canada Ltd) at a dose rate of 1.05 Gy $min^{-1}$. Drug-treated cells were irradiated so that the irradiation was completed 1 h after addition of the drug. X-ray doses of 2.5, 5.0, 10 and 15 Gy were used. A drug concentration of 500 $\mu M$ was used so that there would be only limited toxicity from the 1,2-dithiol-3-thiones and dithioesters.

### *Cell viability measurements*

Cell viability was measured by the ability of single cells to form colonies *in vitro*, as described previously (Teicher *et al.*, 1981, 1985). Following treatment, suspensions of known cell numbers were plated in plastic Petri dishes and allowed to grow in a 37°C incubator under standard culture conditions for 8–10 days. After this time, macroscopic colonies were stained with crystal violet in methanol containing 3.7% formaldehyde and were counted manually. Each experiment was repeated three to five times, and each data point per experiment represents the results of three different dilutions of cells plated in triplicate.

### *Calculated value*

The radioprotection factor (RPF) was calculated as the ratio of the dose of radiation to kill 1 log of normally oxygenated cells with the drug present to dose of radiation without drug to kill 1 log of normally oxygenated cells corrected for the cytotoxicity of each drug.

### Results

In general, 1 h exposure to the substituted 1,2-dithiol-3-thiones and dithioesters had a relatively low level of cytotoxicity toward both normally oxygenated and hypoxic EMT6 cells as measured by the $IC_{50}$ (Table I). The 4-methyl-5-(2-thienyl) analogue (compound *2*) was slightly more cytotoxic towards hypoxic EMT6 cells. The 2-ethoxycarbonyl analogue of compound *2* (compound *3*) and Oltipraz (compound *4*) were 16-fold and 5-fold more cytotoxic toward hypoxic than normally oxygenated EMT6 cells. In contrast, of the dithioesters, only the 3-morpholino-3-(2-thienyl) analogue (compound *11*) was significantly more cytotoxic toward hypoxic EMT6 cells than toward normally oxygenated EMT6 cells.

Radiation survival curves for normally oxygenated and hypoxic EMT6 cells *in vitro* in the presence and absence of 1,2-dithiol-3-thiones are shown in Figure 1. The concentration of each compound used for these radiation studies was 500 μM and the agents were present for 1 h prior to and during radiation delivery. The curves are corrected for the small amount of drug killing. The simple 5-(2-thienyl)-1,2-dithiol-3-thione (compound *1*) was an excellent radiation protector of normally oxygenated EMT6 cells, affording a protection factor of 2.7 at 1 log of cell killing. The 4-methyl derivative of compound *1* (compound *2*) was a much less effective radiation protector, with a protection factor of only 1.2; however, compound *2* increased the cytotoxicity of the radiation treatments in hypoxic EMT6 cells. It appeared that the main effect of compound *2* on the radiation response of hypoxic EMT6 cells was to eliminate the shoulder of the radiation survival curve. The 4-ethoxycarbonyl derivative

**Figure 1** Radiation survival of EMT6 mouse mammary tumour cells in the presence of 1,2-dithiol-3-thiones (compounds *1–4* from Table 1). No drug, normally oxygenated cells (●); no drug, hypoxic cells (○); 500 μM drug, normally oxygenated cells (■); 500 μM drug, hypoxic cells (□). The data are presented as means ± SEM (bars) for three independent experiments.

(compound *3*) produced a good level of radiation protection in normally oxygenated EMT6 cells, with a protective factor of 1.7. Compound *3*, like compound *1*, had no effect on the radiation survival of hypoxic EMT6 cells. The 4-methyl-5-(2-pyrazinyl)-1,2-dithiol-3-thione (compound *4*; Oltipraz), like the other 4-methyl derivative, compound *2*, increased the killing of hypoxic EMT6 cells by radiation. The main effect of compound *4* in hypoxic EMT6 cells also appeared to be elimination of the shoulder of the radiation survival curve, although there was a small increase in the slope of the hypoxic radiation survival curve as well.

Radiation survival curves of normally oxygenated and hypoxic EMT6 cells in the presence and absence of dithioesters are shown in Figure 2. Methyl 3-(1-pyrrolidino)-2-phenylpropenedithiocarboxylate (compound *6*) was an excellent radioprotector of normally oxygenated EMT6 cells, with a protection factor of 2.6, but had no effect on the radiation survival of hypoxic EMT6 cells. Methyl 3-(1-piperidino)-2-phenylpropenedithiocarboxylate (compound *7*) was also an excellent radiation protector of normally oxygenated EMT6 cells, with a radiation protection factor of 2.7, but again there was no significant effect on the radiation

**Figure 2** Radiation survival of EMT6 mouse mammary tumour cells in the presence of dithioesters (compounds *6–9* from Table 1). No drug, normally oxygenated cells (●); no drug, hypoxic cells (○); 500 μM drug, normally oxygenated cells (■); 500 μM drug, hypoxic cells (□). The data are presented as means ± SEM (bars) for three independent experiments.

survival of hypoxic EMT6 cells. The 3-morpholino derivative (compound *8*) similarly produced excellent radiation protection in normally oxygenated EMT6 cells, with a protection factor of 2.6. Unlike compounds *6* and *1*, compound *8* increased the cytotoxicity of radiation towards hypoxic EMT6 cells to a small extent. The 3-thiomorpholino derivative (compound *9*) was the most effective radioprotector of normally oxygenated EMT6 cells tested, with a protection factor of 2.9, and compound *9* did not alter the radiation response of hypoxic EMT6 cells.

Radiation survival curves for normally oxygenated and hypoxic EMT6 cells in the presence and absence of the remaining compounds are shown in Figure 3. Compound *5*, the salt formed by the reaction of methyl iodide with the corresponding 1,2-dithiol-3-thione, was a highly effective radioprotective agent of normally oxygenated EMT6 cells, with a protection factor of 2.6 Compound *5* had no effect on the radiation survival of hypoxic EMT6 cells. Methyl 3-cyclohexylamino-2-phenylpropenedithiocarboxylate (compound *10*) also had a highly effective radiation protection factor of

**Figure 3** Radiation survival of EMT6 mouse mammary tumour cells in the presence of a 1,2-dithiolium salt (compound *5* from Table 1) and of substituted dithioesters (compounds *10–12* from Table 1). No drug, normally oxygenated cells (●); no drug, hypoxic cells (○); 500 μM drug, normally oxygenated cells (■); 500 μM drug, hypoxic cells (□). The data are presented as means ± SEM (bars) for three independent experiments.

2.6 while producing no change in the radiosensitivity of hypoxic EMT6 cells. Methyl 3-morpholino-3-(2-thienyl)-2-methylpropenedithiocarboxylate (compound *11*) was an effective radiation protective agent, with a radioprotective factor of 2.0, but again did not alter the radiation sensitivity of hypoxic EMT6 cells. Methyl 3-morpholino-3-(2-pyrazinyl)-2-methylpropenedithiocarboxylate (compound *12*) was a moderately effective radioprotective agent, with a resulting radiation protection factor of 1.6 However, this compound also increased the radiosensitivity of hypoxic EMT6 cells, although to a lesser extent.

### Discussion

1,2-Dithiol-3-thiones have demonstrated chemoprotective actions against a wide variety of structurally diverse carcinogens (Wattenberg & Bueding, 1986; Kensler *et al.*, 1987; Helmes *et al.*, 1989). These compounds have been used medicinally as antischistosomal agents, choleretics, and to stimulate salivary secretion (Lozac'h & Stavaux, 1980; Bueding *et al.*, 1982; Hausler, 1979; Archer, 1985).

We have examined the radioprotective potential of four 1,2-dithiol-3-thiones and seven dithioesters which are chemically derived from dithiolthiones. Two of the 1,2-dithiol-3-thiones were effective radioprotectors of normally oxygenated EMT6 cells and two showed very little radioprotective activity. Both ineffective compounds had 4-methyl substituents and both of these agents increased the radiation sensitivity of hypoxic EMT6 cells quite markedly. It may be that having a hydrogen in the 4-position allows ring opening of the 1,2-dithiol-ring to occur providing a substantial increase in non-protein sulphydryl within cells and thus radioprotection. A methyl group in the 4-position, on the other hand, may block the ring opening, leading to an inactive molecule. The mechanism for the hypoxic cell selectivity of compounds *3* and *4* remains to be elucidated. The dithioesters were, in general, less cytotoxic than the 1,2-dithiol-3-thiones but also highly effective radiation protective agents. It may be envisioned that oxidative metabolism of the dithioesters could lead to the release of a thiomethylating species, a potent radical scavenger.

Since most normal tissues are well oxygenated, it is the normally oxygenated EMT6 cells which are the most appropriate model for a radioprotector in a cell culture system. Our work with these agents is continuing and we are examining the efficacy of selected examples as normal tissue radiation protectors *in vivo*.

This work was supported by grants nos. PO1 CA38493 and PO1 CA19589 from the National Cancer Institute.

### References

ANSHER, S.S., DOLAN, P. & BUEDING, E. (1986). Biochemical effects of dithiol-thiones. *Food Chem. Toxicol.*, **25**, 405.

ARCHER, S. (1985). The chemotherapy of schistosomiasis. *Ann. Rev. Pharmacol. Toxicol.*, **25**, 485.

BUEDING, E., DOLAN, P. & LEROY, J.P. (1982). The antischistosomal activity of Oltipraz. *Res. Commun. Chem. Pathol. Pharmacol.*, **37**, 293.

CONSTINE, L.S., ZAGERS, G., RUBIN, P. & KLIGERMAN, M. (1986). Protection by WR-2721 of human bone marrow function following irradiation. *Int. J. Radiat. Oncol. Biol. Phys.*, **12**, 1505.

DAVIES, M.H., BLACKER, A.M. & SCHNELL, R.C. (1987). Dithiolthione-induced alterations in hepatic glutathione and related enzymes in male mice. *Biochem. Pharm.*, **36**, 568.

DE LONG, M.J., DOLAN, P., SANTAMARIA, A.B. & BUEDING, E. (1986). 1,2-Dithiol-3-thione analogs: effects on NAD(P)H: quinone reductase and glutathione levels in murine hepatoma cells. *Carcinogenesis*, **7**, 977.

FOYE, W.O., JONES, R.W. & GHOSHAL, P.K. (1987). Antiradiation compounds XXII. Methyl 3-amino-2-phenyldithiopropenoates and 1,1-*bis*(methylthio)-3-amino-2-phenyl-1-propenes. *J. Pharm. Sci.*, **76**, 809.

GLOVER, D., GRABELSKY, S., FOX, K., WEILER, C., CANNON, L. & GLICK, J. (1989). Clinical trials of WR-2721 and cis-platinum. *Int. J. Radiat. Oncol. Biol. Phys.*, **16**, 1201.

HAUSLER, R. (1979). Clinical study with a sialagogue drug (Sulfarlem S 25 = TTPT) in the treatment of xerostomia. *Rev. Suisse Praxis Med.*, **68**, 1063.

HELMES, C.T., BECKER, R.A., SEIDENBERG, J.M., SCHINDLER, J.E. & KELLOFF, G. (1989). Chemoprevention of mouse skin tumorigenesis by dietary Oltipraz. *Proc. Am. Assoc. Cancer Res.*, **30**, 177.

KENSLER, T.W., EGNER, P.A., DOLAN, P.M., GROOPMAN, J.D. & ROEBUCK, B.D. (1987). Mechanism of protection against the tumorigenicity in rats fed 5-(2-pyrazinyl)-4-methyl-1,2-dithiol-3-thione (Oltipraz) and related 1,2-dithiol-3-thiones and 1,2-dithiol-3-thiones. *Cancer Res.*, **47**, 4271.

LOZAC'H, N. & STAVAUX, M. (1980). The 1,2 and 1,3-dithiolium ions. *Adv. Heterocycl. Chem.*, **27**, 151.

ROCKWELL, S.C. & KALLMAN, R.F. (1973). Cellular radiosensitivity and tumor radiation response to the EMT6 tumor cell system. *Radiat. Res.*, **53**, 281.

ROCKWELL, S.C., KALLMAN, R.F. & FAJARDO, L.F. (1972). Characteristics of serially transplanted mouse mammary tumor and its tissue culture adapted derivative. *J. Natl Cancer Inst.*, **49**, 735.

TEICHER, B.A., CRAWFORD, J.M., HOLDEN, S.A. & 4 others (1988). Glutatione monoethyl ester can selectively protect liver from high dose BCNU or cyclophosphamide. *Cancer*, **62**, 1275.

TEICHER, B.A. & HOLDEN, S.A. (1987). Antitumor and radiosensitizing activity of several platinum-positively charged dye complexes. *Radiat. Res.*, **109**, 58.

TEICHER, B.A., LAZO, J.S. & SARTORELLI, A.C. (1981). Classification of antineoplastic agents by their selective toxicities toward oxygenated and hypoxic tumor cells. *Cancer Res.*, **41**, 73.

TEICHER, B.A., ROCKWELL, S.C. & LEE, J.B. (1985). Radiosensitivity by nitroaromatic Pt(II) complexes. *Int. J. Radiat. Oncol. Biol. Phys.*, **11**, 937.

WATTENBERG, L.W. & BUEDING, E. (1986). Inhibitory effects of 5-(2-pyrazinyl)-4-methyl-1,2-dithiol-3-thione (Oltipraz) on carcinogenesis induced by benzo(a)pyrene, diethylnitrosamine and uracil mustard. *Carcinogenesis*, **7**, 1379.

YUHAS, J.M., DAVIS, M.E., GLOVER, D., BROWN, D. & RITTER, M. (1982). Circumvention of the tumor membrane barrier to WR-2721 absorption by reduction of drug hydrophilicity. *Int. J. Radiat. Oncol. Biol. Phys.*, **8**, 519.

YUHAS, J.M. & STORER, J.B. (1969). Differential chemoprotection of normal and malignant tissues. *J. Natl Cancer Inst.*, **42**, 331.

YUHAS, J.M., YURCONIC, M., KLIGERMAN, M.M., WEST, G. & PETERSON, D.F. (1977). Combined use of radioprotective and radiosensitizing drugs in experimental radiotherapy. *Radiat. Res.*, **70**, 443.


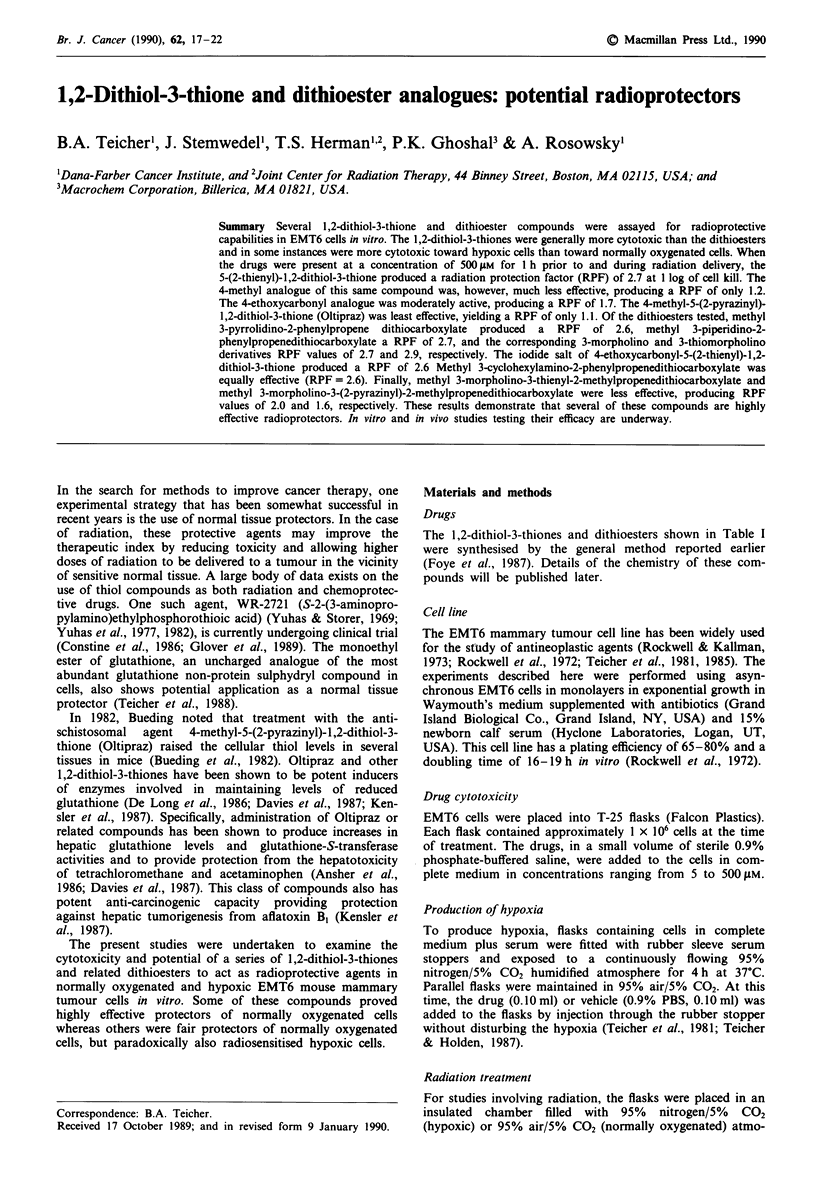

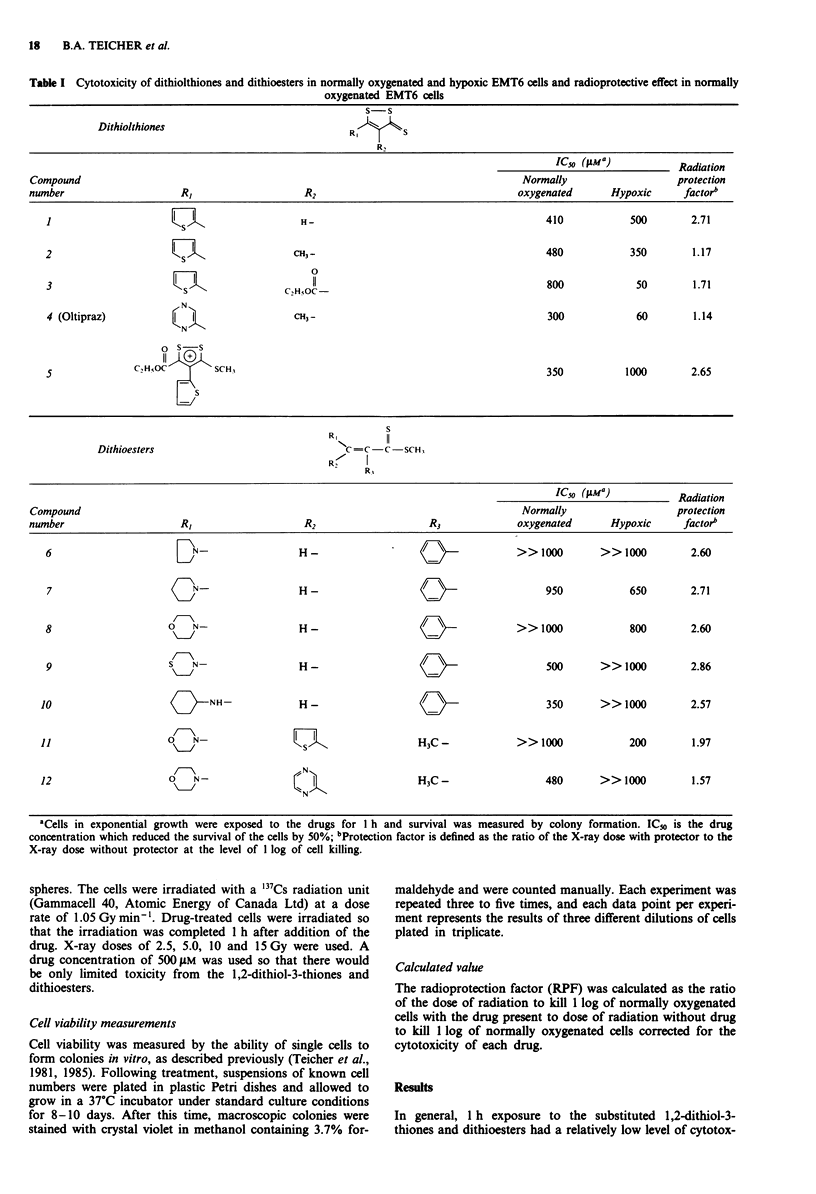

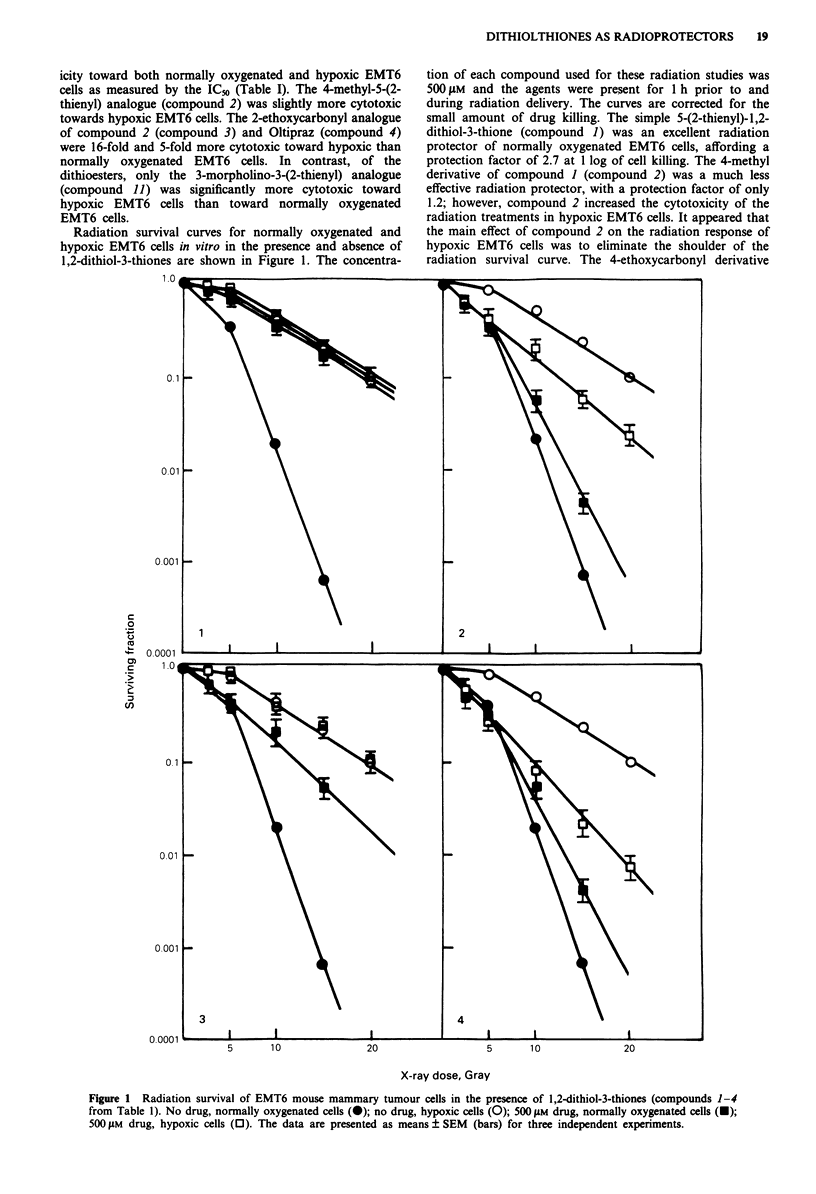

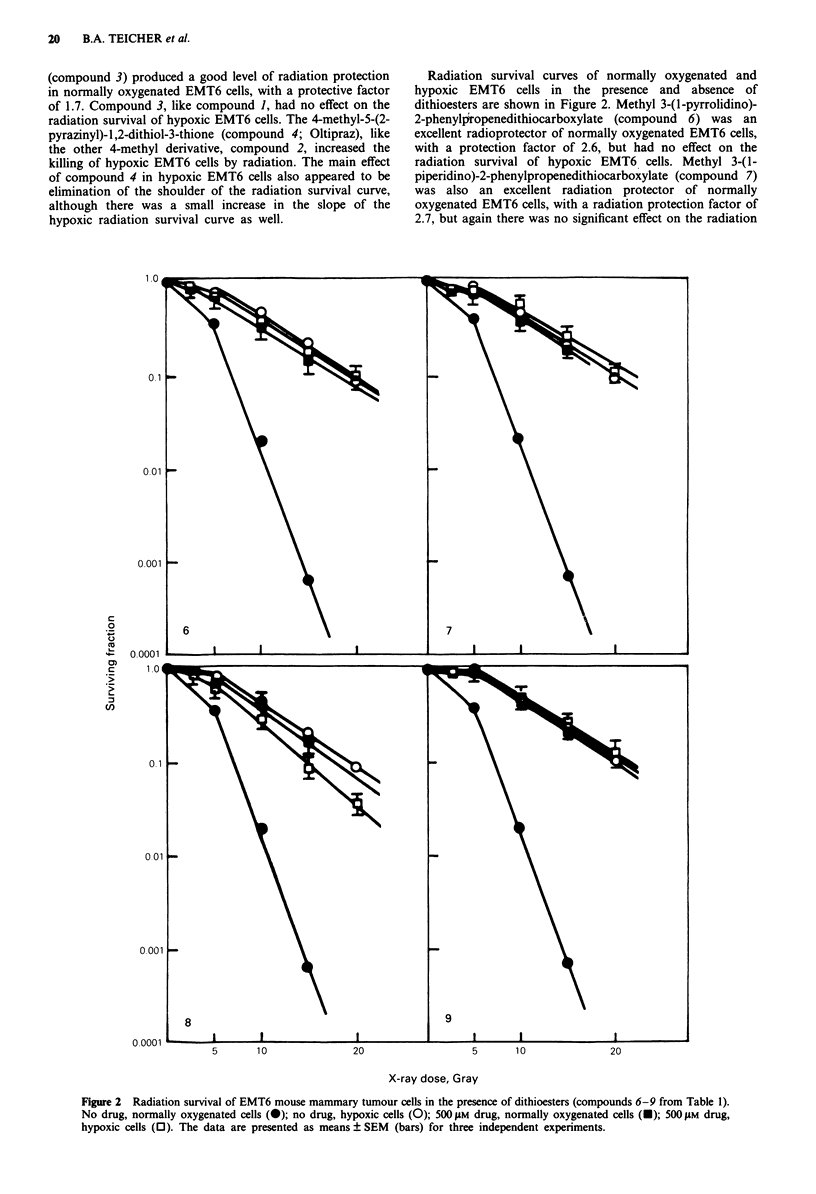

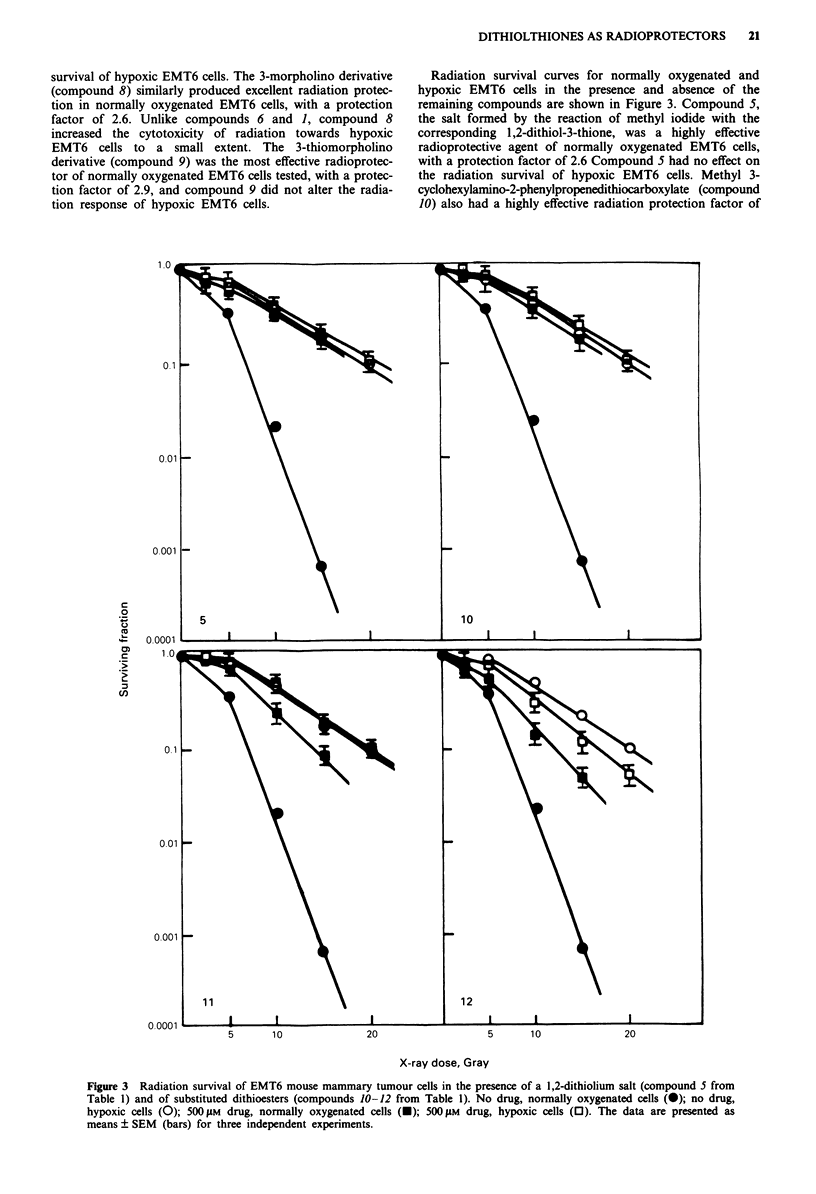

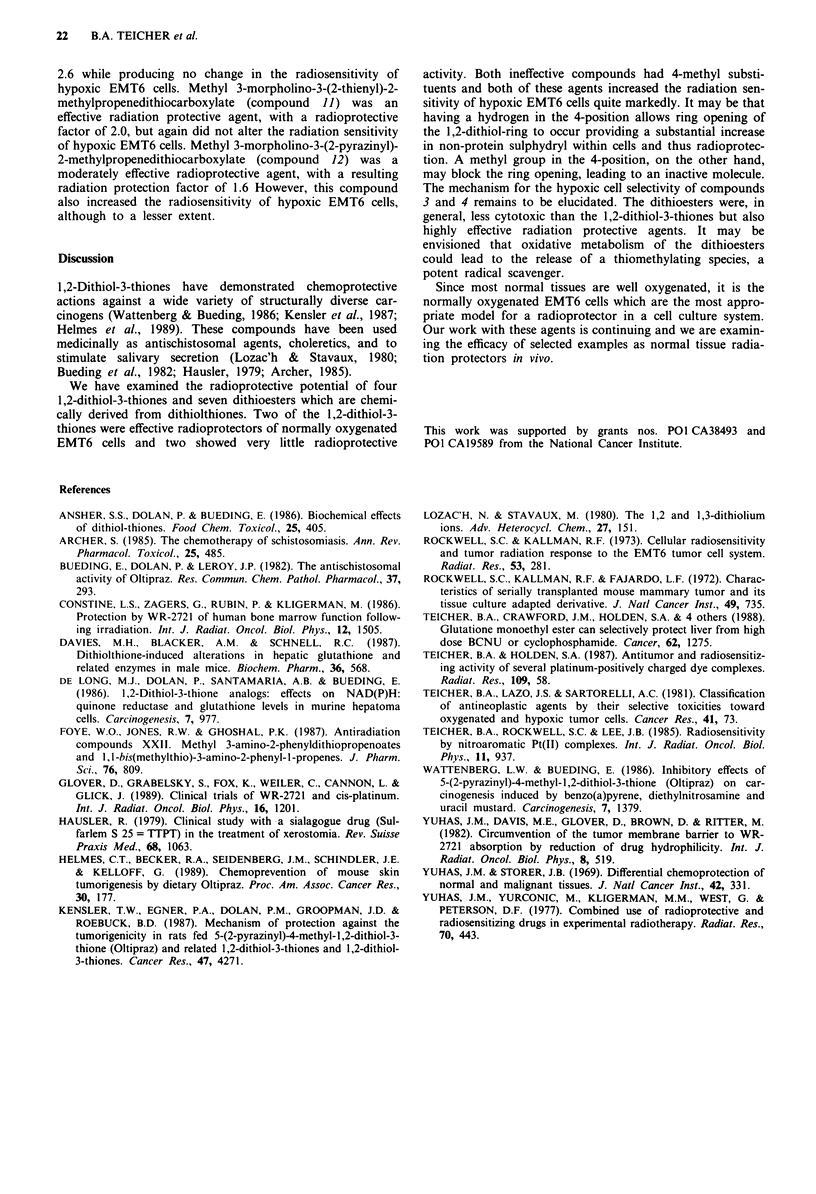


## References

[OCR_00446] Ansher S. S., Dolan P., Bueding E. (1986). Biochemical effects of dithiolthiones.. Food Chem Toxicol.

[OCR_00450] Archer S. (1985). The chemotherapy of schistosomiasis.. Annu Rev Pharmacol Toxicol.

[OCR_00454] Bueding E., Dolan P., Leroy J. P. (1982). The antischistosomal activity of oltipraz.. Res Commun Chem Pathol Pharmacol.

[OCR_00459] Constine L. S., Zagars G., Rubin P., Kligerman M. (1986). Protection by WR-2721 of human bone marrow function following irradiation.. Int J Radiat Oncol Biol Phys.

[OCR_00464] Davies M. H., Blacker A. M., Schnell R. C. (1987). Dithiolthione-induced alterations in hepatic glutathione and related enzymes in male mice.. Biochem Pharmacol.

[OCR_00469] De Long M. J., Dolan P., Santamaria A. B., Bueding E. (1986). 1,2-Dithiol-3-thione analogs: effects on NAD(P)H:quinone reductase and glutathione levels in murine hepatoma cells.. Carcinogenesis.

[OCR_00475] Foye W. O., Jones R. W., Ghoshal P. K. (1987). Antiradiation compounds. XXII. Methyl 3-amino-2-phenyldithiopropenoates and 1,1-bis(methylthio)-3-amino-2-phenyl-1-propenes.. J Pharm Sci.

[OCR_00481] Glover D., Grabelsky S., Fox K., Weiler C., Cannon L., Glick J. (1989). Clinical trials of WR-2721 and cis-platinum.. Int J Radiat Oncol Biol Phys.

[OCR_00486] Häusler R., Ritschard J. (1979). Utilisation d'un sialagogue (sulfarlem S 25 = TTPT) dans le traitement de la xérostomie.. Schweiz Rundsch Med Prax.

[OCR_00497] Kensler T. W., Egner P. A., Dolan P. M., Groopman J. D., Roebuck B. D. (1987). Mechanism of protection against aflatoxin tumorigenicity in rats fed 5-(2-pyrazinyl)-4-methyl-1,2-dithiol-3-thione (oltipraz) and related 1,2-dithiol-3-thiones and 1,2-dithiol-3-ones.. Cancer Res.

[OCR_00513] Rockwell S. C., Kallman R. F., Fajardo L. F. (1972). Characteristics of a serially transplanted mouse mammary tumor and its tissue-culture-adapted derivative.. J Natl Cancer Inst.

[OCR_00508] Rockwell S., Kallman R. F. (1973). Cellular radiosensitivity and tumor radiation response in the EMT6 tumor cell system.. Radiat Res.

[OCR_00517] Teicher B. A., Crawford J. M., Holden S. A., Lin Y., Cathcart K. N., Luchette C. A., Flatow J. (1988). Glutathione monoethyl ester can selectively protect liver from high dose BCNU or cyclophosphamide.. Cancer.

[OCR_00522] Teicher B. A., Holden S. A. (1987). Antitumor and radiosensitizing activity of several platinum-(+) dye complexes.. Radiat Res.

[OCR_00527] Teicher B. A., Lazo J. S., Sartorelli A. C. (1981). Classification of antineoplastic agents by their selective toxicities toward oxygenated and hypoxic tumor cells.. Cancer Res.

[OCR_00532] Teicher B. A., Rockwell S., Lee J. B. (1985). Radiosensitization of EMT6 cells by four platinum complexes.. Int J Radiat Oncol Biol Phys.

[OCR_00537] Wattenberg L. W., Bueding E. (1986). Inhibitory effects of 5-(2-pyrazinyl)-4-methyl-1,2-dithiol-3-thione (Oltipraz) on carcinogenesis induced by benzo[a]pyrene, diethylnitrosamine and uracil mustard.. Carcinogenesis.

[OCR_00543] Yuhas J. M., Davis M. E., Glover D., Brown D. Q., Ritter M. (1982). Circumvention of the tumor membrane barrier to WR-2721 absorption by reduction of drug hydrophilicity.. Int J Radiat Oncol Biol Phys.

[OCR_00549] Yuhas J. M., Storer J. B. (1969). Differential chemoprotection of normal and malignant tissues.. J Natl Cancer Inst.

[OCR_00553] Yuhas J. M., Yurconic M., Kligerman M. M., West G., Peterson D. F. (1977). Combined use of radioprotective and radiosensitizing drugs in experimental radiotherapy.. Radiat Res.

